# When Foreign Body Aspiration Is Not the Whole Story: Reconsidering Imaging Escalation in Infants With Apparent Tension Pneumothorax

**DOI:** 10.1002/ccr3.72805

**Published:** 2026-05-28

**Authors:** Muhammad Abdullah Awan

**Affiliations:** ^1^ Wah Medical College National University of Medical Sciences Rawalpindi Pakistan

**Keywords:** bronchoscopy, computed tomography, congenital pulmonary airway malformation, foreign body aspiration, pediatric airway emergency, tension pneumothorax

## Abstract

In infants with suspected foreign body aspiration and radiographic tension pneumothorax, failure to improve after appropriate decompression should prompt immediate diagnostic reconsideration. Early CT or advanced imaging may identify congenital lung anomalies such as CPAM, particularly when positive‐pressure ventilation creates misleading radiographic findings.

Dear Editor,

I read with great interest the case report by Ibrahımoglu et al. titled “When Foreign Body Aspiration Is Not the Whole Story: An Undiagnosed Congenital Pulmonary Airway Malformation Mimicking Tension Pneumothorax in an Infant,” published in Clinical Case Reports [1]. The authors describe a clinically important case of an 11‐month‐old infant in whom suspected foreign body aspiration and apparent tension pneumothorax were ultimately complicated by an undiagnosed congenital pulmonary airway malformation (CPAM). This report is valuable because it highlights how an acute pediatric airway emergency may conceal an underlying congenital lesion that substantially alters physiology and interpretation of imaging.

One of the strongest clinical lessons from this case is that initial emergency management may be appropriate even when the final diagnosis is different. In an infant presenting with cardiopulmonary arrest, mediastinal shift, and severe respiratory compromise, immediate decompression for suspected tension pneumothorax is understandable. However, the absence of meaningful clinical improvement after tube thoracostomy became the crucial turning point. Such a discordant response should prompt clinicians to rapidly reassess the working diagnosis rather than persist with the initial interpretation.

The case also emphasizes an important radiological pitfall. CPAM may produce marked hyperlucency and mediastinal shift, especially when air trapping is worsened by positive‐pressure ventilation. In this setting, the radiographic appearance can mimic tension pneumothorax even when the underlying process is not pleural air. The authors' CT findings clarified the diagnosis by demonstrating a large multicystic lesion in the left hemithorax together with a foreign body in the right main bronchus [1]. This sequence illustrates the value of advanced imaging when the clinical course does not match the expected response to emergency intervention.

Another important point is that foreign body aspiration and CPAM should not be viewed as mutually exclusive diagnoses. In the reported infant, the aspirated peanut was the acute precipitating event, while the previously silent CPAM reduced pulmonary reserve and likely amplified hypoxia [1]. This interaction is clinically significant because an otherwise compensated congenital lung malformation may become life‐threatening when the contralateral airway is obstructed. In similar cases, persistent instability after airway or pleural intervention should raise suspicion for a second pathology.

This report also supports a practical diagnostic approach in pediatric emergencies. Physiological stabilization must remain the first priority. However, once the child is stabilized or when the response to initial decompression is inadequate, early escalation to CT, bronchoscopy, or other advanced diagnostic tools should be considered. Bedside ultrasound may also have a role in differentiating pneumothorax from cystic intrapulmonary pathology, depending on local expertise and availability.

The authors' management sequence further demonstrates the importance of multidisciplinary decision‐making. Bronchoscopic removal of the foreign body addressed the reversible airway obstruction, while definitive CPAM resection was appropriately delayed until the child was sufficiently stabilized [1]. This staged approach is clinically reasonable in complex pediatric emergencies, where immediate surgery may increase risk if neurological and respiratory status remain unstable.

From a broader perspective, the case raises the need for diagnostic flexibility in infants with apparent tension pneumothorax after foreign body aspiration. Radiographic hyperlucency, mediastinal shift, and respiratory collapse are alarming findings, but the diagnosis should be reconsidered when chest decompression fails to produce expected improvement. Early recognition of such diagnostic complexity may prevent unnecessary persistence with ineffective interventions and allow more timely identification of congenital lung anomalies.

## Conclusion

This case provides an important reminder that foreign body aspiration may not be the whole explanation in infants with severe respiratory collapse. When apparent tension pneumothorax fails to improve after appropriate decompression, clinicians should promptly reconsider the diagnosis and evaluate for mimicking conditions such as CPAM. Early imaging escalation, bronchoscopy, and multidisciplinary management may improve decision‐making in complex pediatric airway emergencies.

## Author Contributions

**Muhammad Abdullah Awan:** conceptualization, formal analysis, writing – original draft, writing – review and editing.

## Funding

The author has nothing to report.

## Ethics Statement

The author has nothing to report.

## Consent

The author has nothing to report.

## Conflicts of Interest

The author declares no conflicts of interest.

## Data Availability

Data sharing not applicable to this article as no datasets were generated or analysed during the current study.
